# Pharmacological Strategies for Decreasing Opioid Therapy and Management of Side Effects from Chronic Use

**DOI:** 10.3390/children5120163

**Published:** 2018-12-05

**Authors:** Genevieve D’Souza, Anava A. Wren, Christina Almgren, Alexandra C. Ross, Amanda Marshall, Brenda Golianu

**Affiliations:** 1 Department of Anesthesia, Perioperative and Pain Medicine, Stanford University, Palo Alto, CA 94304, USA; gdsouza@stanford.edu (G.D.); calmgren@stanfordchildrens.org (C.A.); alexandra.cram.ross@gmail.com (A.C.R.); 11am203@queensu.ca (A.M.); 2 Department of Pediatrics, Division of Pediatric Gastroenterology, Hepatology and Nutrition, Stanford University, Palo Alto, CA 94304, USA; awren2@stanford.edu

**Keywords:** opioid therapy, weaning of opioids, withdrawal, assessment of withdrawal

## Abstract

As awareness increases about the side effects of opioids and risks of misuse, opioid use and appropriate weaning of opioid therapies have become topics of significant clinical relevance among pediatric populations. Critically ill hospitalized neonates, children, and adolescents routinely receive opioids for analgesia and sedation as part of their hospitalization, for both acute and chronic illnesses. Opioids are frequently administered to manage pain symptoms, reduce anxiety and agitation, and diminish physiological stress responses. Opioids are also regularly prescribed to youth with chronic pain. These medications may be prescribed during the initial phase of a diagnostic workup, during an emergency room visit; as an inpatient, or on an outpatient basis. Following treatment for underlying pain conditions, it can be challenging to appropriately wean and discontinue opioid therapies. Weaning opioid therapy requires special expertise and care to avoid symptoms of increased pain, withdrawal, and agitation. To address this challenge, there have been enhanced efforts to implement opioid-reduction during pharmacological therapies for pediatric pain management. Effective pain management therapies and their outcomes in pediatrics are outside the scope of this paper. The aims of this paper were to: (1) Review the current practice of opioid-reduction during pharmacological therapies; and (2) highlight concrete opioid weaning strategies and management of opioid withdrawal.

## 1. Introduction

There is growing public concern regarding the use and risks related to prescription opioid therapy [1]. Historically, opioid therapy has been a central component to clinical pain management in most pediatric hospitals. Opioids are frequently prescribed for the management of pain related to acute conditions, as well as for sedation and management of agitation among patients receiving intensive care. Research in both adults and children has demonstrated that opioids are effective and potent in the acute management of pain [2,3]. Opioids increase descending modulation, and reduce descending facilitation in pain pathways of the central nervous system [4]. Opioids also target affective centers of the brain that impact emotional states, which can alter pain perception [5].

With long term use, however, physiological dependence and tolerance increase due to internalization of receptors and other adaptive processes [6]. Efficacy of clinical pain control decreases requiring increasing doses of analgesic agents, corresponding to increases in side effect profiles [6]. The range of side effects include respiratory depression, constipation, cognitive dysfunction, and psychiatric comorbidities such as anxiety and depression [7,8,9,10]. Additionally, chronic opioid use can lead to physical tolerance and dependence, and carries the risk of addiction [11]. Opioid use can also have the unexpected result of increased sensitization to pain (i.e., opioid induced hyperalgesia), though this is a rare phenomenon [12].

Understanding the appropriate use and side effect profiles of opioids, and effective tapering strategies, is vital to delivering safe pain management to pediatric patients. A search in Pubmed was performed on the following topics in both pediatric and adult literature: Opioid dependence, tolerance, withdrawal, and weaning from 2000 to 2018. Articles focused on pediatric data were chosen. Adult data was reviewed and utilized when pediatric data was lacking. Where data was pediatric-focused, specific references were made in the text. All other references refer to adult data.

The aims of this paper were to: (1) Review the current practice of opioid-reduction during pharmacological therapies; and (2) highlight concrete opioid weaning strategies, and management of opioid withdrawal. We reviewed and defined opioid dependence, withdrawal, tolerance and addiction, assessment of opioid withdrawal utilizing age appropriate withdrawal scales, and utilization of wean strategies and pharmacological aids to assist in safe reduction in therapy. This article did not address the use of opioids for pain management specifically or aim to assess pain management outcomes of opioid therapy. 

## 2. Opioid-Reduction Weaning Protocols in Pediatric Patients

Effective medical management of patients receiving opioids can be addressed by appropriate assessment of pain, an accurate understanding of opioids and related side effects and withdrawal, and implementation of opioid reduction strategies including opioid weaning protocols and opioid-sparing adjuvants. Opioid withdrawal may occur in 0–100% of children who are exposed to opioids. Prevalence ranged from 45 to 86% in one recent review [13], while in another study 68% of patients experienced withdrawal [14].

Pediatric weaning protocols are highly variable. In a recent survey of pediatric healthcare providers, only 27% of respondents utilized a written protocol for opioid tapering, 22% consulted a pain management team regarding weaning of opioids, and the majority seldom or never consulted a pharmacist [15]. In another survey of pediatric congenital heart disease centers, only 25% of sites used a standardized clinical pathway when weaning opioid medications [16]. In the RESTORE study—a randomized controlled multicenter study of protocolized sedation of 1225 pediatric patients—Curley et al. demonstrated that the use of protocolized weaning of opioid medication was associated with shorter durations of opioid treatment (9 vs. 10 days, *p* = 0.01), and more study days spent in the calm and awake states (86% vs. 75%, *p* = 0.004) [14].

Common management strategies described in small pediatric studies include the use of methadone, addition of alpha 2 agonists, and protocolized reduction in dosing [17]. The most common weaning strategies involved decreases of 10–20% per day [13]. Identification of high-risk patients can help prevent iatrogenic withdrawal [18]. These include young age, pre-existing cognitive impairment, higher mean preweaning opioid dose, duration of opioid treatment, use of three or more sedative medications, and higher nurse to patient ratio.

The developmental aspects of opioid therapy deserve some consideration. The endogenous opioid system is highly expressed by developing neural cells, and can influence both neuronal as well as glial maturation [19]. While opioids do not directly trigger maturational events, they modulate ongoing cellular processes. These potentially detrimental effects must be balanced against the effects of untreated pain and physiologic stress, which have been associated with adverse outcomes and may also affect subsequent development [20], as well as ongoing clinical care of critically ill children [21]. Research on long-term neurocognitive effects of opioids on children is limited, and confounded by multiple co-existing morbidities [22].

The use of synthetic or short acting opioids may cause greater tolerance in preterm and term neonates. For example, patients receiving fentanyl while on extracorporeal membrane oxygenation (ECMO) require longer durations of opioid weaning compared to neonates who have received morphine during their treatment [23]. Additionally, neonates have immature kidney and liver functions leading to the accumulation of morphine metabolites, notably morphine-3-glucuronide, which can lead to antagonism of analgesia, as well as morphine-6-glucuronide, which is a more potent analgesic in comparison to morphine and may lead to excessive sedation [24]. 

Therefore, the treating physician must be aware of developmental concerns from pharmacokinetic to pharmacodynamic perspectives, as well as the developmental needs of the child from a neurological and psychological perspective at each phase of the treatment, and weaning processes. The use of appropriate and well timed adjuvant therapies, both pharmacological and nonpharmacological, may help minimize the need for sedatives and analgesics at various stages of the treatment process [25].

## 3. Opioids and Common Side Effects

The mechanisms of opioid analgesia include inhibiting the transmission of noxious input from the periphery to the central nervous system, and facilitating the descent of the inhibitory system that can decrease noxious input and affective modulation [26,27]. While opioids are very effective at the acute management of pain, some pediatric patients develop physiologic dependence or withdrawal following as little as seven days of opioid therapy [28]. Tolerance and addiction are other potential physiological effects of opioids. See Table 1 for a summary of potential physiological effects described below.

### 3.1. Dependence and Withdrawal

Dependence is an involuntary physiological response that occurs when there is an adaptation to the opioid, which is manifested by withdrawal symptoms when the opioid is abruptly discontinued [30,32]. Common neurologic signs of dependence in children include anxiety, agitation, grimacing, insomnia, increased muscle tone, abnormal tremors, and choreoathetoid movements. Gastrointestinal symptoms include vomiting, diarrhea, and poor appetite, while autonomic signs include tachypnea, tachycardia, fever, sweating, and hypertension [21].

In children, there are still many unanswered questions regarding the long term effects of opioid tolerance and withdrawal [33]. Sirnes et al., in a preliminary study, found that school-age children who had been exposed to prenatal opioids, showed Functional Magnetic Resonance Imaging (fMRI) changes and possible impaired executive function task performance [34]. Although some preliminary animal data suggests that there may be a faster onset of dependence in younger rats, it is unclear whether this applies to pediatric patients, and more studies are needed in this area [21].

The onset of withdrawal is dependent on the characteristics of the opioid the child was exposed to. For shorter acting opioids, such as oxycodone or hydrocodone, the symptoms may start as soon as 12 h after the last dose and peak over the next one or two days. In contrast, when stopping a longer acting opioid such as methadone, the signs of withdrawal may not be seen for more than 24 h and can last up to 10 days [35].

Studies have shown that children at increased risk of iatrogenic withdrawal syndrome include those who are less than six months old, have had a higher cumulative opioid dose or longer duration of exposure, have been exposed to three or more sedative classes (e.g., opioids, benzodiazepines, dexmedetomidine, and ketamine), or have a pre-existing cognitive impairment [18]. Of note, in neonatal abstinence syndrome, newborn infants may be dependent on opioids or benzodiazepines due to in-utero exposure. Following birth, they must be closely observed for signs of withdrawal, and treated as appropriate [18].

In older children, symptoms of delirium—a neurocognitive disorder due to a somatic illness or its treatment—can mimic those of withdrawal such as tachycardia, tachypnea, agitation, irritability, muscle tension, and sleep disturbances [36]. Children who are critically ill or in the Intensive Care Unit (ICU) are at risk of delirium secondary to the multiple sedative agents used, the severity of illness, changes in metabolic dysfunction or infection. Environmental factors such as changes in sleep can be additive [37,38]. One study found that children who received benzodiazepines were at a five-times greater risk of developing delirium than those that did not receive benzodiazepines [39]. Several delirium scoring tools have been developed, such as the Pediatric Confusion Assessment Method for ICU in children over five years of age (modified version of the adult assessment tool) [37]. Several symptoms of delirium such as tachycardia, tachypnea, and agitation are scored on both the withdrawal and delirium scales making definitive diagnostic determination difficult. Appropriate treatment of delirium may include, but is notlimited to, the treatment of any underlying withdrawal.

### 3.2. Tolerance

Tolerance is a state of physiological adaptation that occurs after prolonged exposure to an opioid. With tolerance, the patient requires progressively larger doses of the opioid to achieve the same effect. Complex intracellular neural mechanisms, including opioid receptor desensitization and down-regulation, are believed to be key mechanisms underlying opioid tolerance [40]. Tolerance can occur as soon as three days after opioid exposure [41]. In critically ill children, the effects can be profound. One multicenter study found that 16% of mechanically ventilated children required doubling of their opioid doses after seven days, and 20% doubled their requirement after 14 days [42]. In spite of potentially rapid development of tolerance in the pediatric population, especially in the case of life-limiting illnesses such as oncologic conditions or other severe illnesses, the rapid escalation and resultant tolerance to keep severely ill pediatric patients comfortable is often justified. [43,44].

### 3.3. Opioid Induced Hyperalgesia

Research has shown that the administration of higher doses of opioids over longer periods of time does not always lead to improved pain control, rather it can result in hyperalgesia. A recent study found that the regular use of opioids for more than 90 days led to increased pain sensitivity [45]. The exact mechanisms by which patients developcentral sensitization found in opioid induced hyperalgesia is still unknown, but possible contributing factors include characteristics of the opioids being used, changes in corticotrophin releasing factors, suppressed reuptake or increased release of excitatory neurotransmitters, changes in glial cells, and genetic variability [46,47]. Opioid induced hyperalgesia (OIH) is less commonly addressed in pediatric literature. One case report from 2012 noted the development of OIH in a nine year-old receiving opioids for flare of polyarticular juvenile idiopathic arthritis. The child was treated by stopping the opioid. Subsequently, pain and hyperalgesia decreased [48]. Treatment for OIH often involves the reduction or elimination of the opioid to monitor for resolution of symptoms. Ongoing adult research is evaluating ketamine and methadone, which have NMDA receptor antagonist activity, to determine if these medications decrease the likelihood of developing OIH or help resolve symptoms [12].

### 3.4. Addiction

Chronic opioid use also has the potential to lead to addiction. Addiction, as defined by the American Pain Society and the American Society of Addiction Medicine, is a chronic neurobiological disease with genetic, psychosocial, and environmental factors contributing to its development and presentation [31]. Addiction is characterized by the inability to consistently abstain from an addictive stimulus, impairment in behavioral control, craving, diminished recognition of significant problems with one’s behaviors and interpersonal relationships, and a dysfunctional emotional response.

Research has demonstrated that approximately one in four adults receiving chronic opioid therapy in a primary care setting suffers from opioid addiction [49,50,51]. To our knowledge, there are no published data detailing rates of opioid addiction among youth. Interestingly, in a Canadian study examining methadone maintenance therapy, initial age of opioid use was associated with increased physical and psychological comorbidities. Individuals with an age-of-onset of opioid use younger than 18 years were found to be at higher odds of having a physical or psychiatric comorbid disorder, compared to individuals with an age-of-onset of 31 years or older [52]. Future research should explore rates and risk factors of opioid addiction among pediatric populations, as well as risk factors for addiction. Appropriate treatment of comorbid disorders in patients who are being considered for opioid therapy is recommended to minimize the risk of opioid addiction.

## 4. Assessment of Opioid Withdrawal

Assessment of opioid withdrawal is central to developing and applying effective opioid-reduction during pharmacological treatments. A number of scales have been developed to help providers distinguish between signs of neonatal withdrawal and iatrogenic withdrawal, and to help determine when treatment might be required. The Finnegan neonatal abstinence scale, and its recent modification, allows clinicians to accurately assess clinical symptoms of neonatal withdrawal [53]. Scores >8 indicate the necessity for further evaluation for treatment.

The Withdrawal Assessment Tool-1 (WAT-1) scores youth on symptoms of withdrawal including tremors, increased muscle tone, repetitive movements, loose stools, and vomiting. This tool was originally validated for children six months to eight years of age [32]; however, more recent studies have found WAT-1 to be reliable for youth up to 17 years of age [54].

The Sophia Observation withdrawal Symptoms scale scores infants and children three months or older on withdrawal symptoms within a four-hour period. Scores >4 should be evaluated further for withdrawal. This scale was found to have a sensitivity of 83%, and specificity of 95% in critically ill children in the Pediatric Intensive Care Unit (PICU) [55]. Only the Sophia Observation withdrawal scale and WAT-1 have been specifically developed to evaluate iatrogenic withdrawal in a range of ages and settings [54].

## 5. Opioid Weaning Treatments

Although there is no certainty about whether an individual patient will experience symptoms of withdrawal, some general guidelines are helpful for the clinician to consider in developing an individualized opioid-reduction program.

For opioid therapy lasting less than seven days, no gradual reduction in dose is necessary as patients are at a lower risk of withdrawal. The opioid infusion may be discontinued without a taper, and patients should be monitored for signs and symptoms of withdrawal. Additional factors placing a patient at increased risk for withdrawal following opioid therapy lasting less than seven days include higher cumulative dose of opioids and prior exposure to opioids.

When opioid therapy has been delivered for more than seven days, or symptoms of withdrawal are present, gradual opioid weaning is usually necessary. Opioid weaning protocols aim to gradually decrease plasma concentrations of the drug to prevent symptoms of opioid withdrawal syndrome. As no definite outcome-based evidence exists to support an ideal weaning protocol, therapy should be based on the individual treatment response, and should consider the length of opioid exposure and the total daily opioid dose (TDD) [56]. One suggested weaning schedule is to decrease the TDD by 10–20% everyday, while monitoring for signs and symptoms of withdrawal. Oral morphine, methadone, clonidine, and transdermal fentanyl have all been used in opioid weaning programs.

Methadone is often the most suitable medication for weaning due to the long half-life and convenience of oral dosing [57,58]. To account for cross tolerance, the converted methadone dosage may need to be reduced by 30–50%. The total daily dose of methadone is then divided into four equal doses to be given every 6 h (q6hr). Initial q6hr dosing is for 24 h only—this is functionally a “loading dose” to rapidly increase serum levels of methadone. Due to the long half-life of methadone, the dosing interval will change to every 12 h after the initial 24 h period, with close observation for possible sedation. Once a steady state is reached, methadone can then be tapered by 10–20% every other day, observing for symptoms of withdrawal. Of note, methadone is not suitable for children with prolonged QT corrected (QTc). A baseline EKG is recommended prior to initiating therapy with methadone. In these critically ill children, the TDD of their current opioid may be decreased by 10–20% daily until the wean is accomplished, or the patient may be transitioned to another oral or intravenous opioid (e.g., morphine, hydromorphone). See Table 2 for sample opioid weaning schedules.

Transitioning to enteral methadone should be considered as early as clinically feasible. The bioavailability of enteral methadone is 75–80% that of intravenous methadone, thus it is not usually necessary to adjust dosing for this difference. If converting from high doses of opioid infusions, it is recommended that intravenous infusions be tapered to lower doses (e.g., ≤0.2 mg/kg/h morphine equivalent) before transitioning to methadone.

When weaning patients from opioids, withdrawal and related symptoms often occur and can be distressing for children. Clonidine can be useful in treating these symptoms of opioid withdrawal.

Duffet et al. performed a prospective randomized control trial (RCT) on 50 pediatric patients undergoing reduction of opioid therapy and studied the use of parenteral clonidine 5 µg/kg, administered every 6 h [59]. No significant effect on withdrawal symptoms was observed. Thirteen patients experienced hypotension or bradycardia requiring intervention; however, the relationship to the study group was unclear.

The use of clonidine as a prophylactic for withdrawal syndromes was studied in 10 consecutive patients undergoing single-state laryngotracheal reconstruction [60]. No significant side effects were noted. A study of plasma concentrations of whole vs. cut patches showed that the delivered dose from whole patches was found to be 7 ± 1.7 µg/kg/day (plasma concentration 0.55 ± 0.3 ng/mL), while cut patches delivered a lower dose but with increased variability, 6.4 ± 3 µg/kg/day (plasma concentration 1 ± 1.1 ng/mL) [61]. A recent review suggested that, while oral and transdermal clonidine have a potential role in the management of sedation and withdrawal in critically ill infants and children, and were relatively well tolerated, the use of cut transdermal patches should be avoided [62]. Further prospective studies were recommended. No data exists on the weaning of clonidine dose following its use as an adjuvant for opioid withdrawal [53]. The recommendations noted below are a result of developed clinical practice at our institution.

### Sample Clonidine Dosing and Weaning Schedule

1. Start with clonidine 4–8 µg/kg/day oral (per os (PO)) divided every 4 h. If not taking oral intake (nil per os (NPO)), give clonidine 1 µg/kg IV q4h.
2. Concurrently initiate opioid weaning by reducing daily opioid dose by 5–10% of baseline dose.
3. After 48 h on oral or parenteral clonidine without adverse blood pressure effects, convert to clonidine patch 5–10 µg/kg/day, rounding up or down to nearest 50 µg increment.
4. The minimum patient weight for patch use is typically about 5 kg. Although doses as high as 2 µg/kg may be tolerated, the physical patch size makes it difficult to use in smaller children. Therefore, for children ≤5 kg, use oral or intravenous (i.v.) clonidine.
5. Once ready to wean, reduce the clonidine patch size by 50 µg/day two times a week until the dose is reduced to the 100 µg/day patch size for children >10 kg, or 50 µg/day patch size for children <10 kg. Then leave this last patch on for 14 days and discontinue afterwards.
6. Clonidine weaning may occur concomitantly with opioid weaning, provided the opioid parenteral morphine equivalent dose is less than 0.3 mg/kg/day.

For excessive withdrawal (WAT-1 > 3), the total as needed (pro re nata (PRN)) morphine requirement may be added to the daily methadone dose, and the taper resumed once symptoms are controlled consistently. For excessive sedation, a dose of methadone should be held, and the total daily methadone dose decreased by 10–20%. Ondansetron 0.1 mg/kg up to 4 mg every 6 h may be used to treat nausea related to withdrawal [63]. Patients should be assessed for QT prolongation, particularly if other QT-prolonging medications are being used [64]. Co-administration of benzodiazepines with opioids is frequently practiced in the context of intensive care sedation or in acute postoperative pain management settings. When discontinuation is planned, the benzodiazepines may need concurrent weaning, often alternating with the opioid wean, as described in a recent review by Fenn et al. [13]. Care should be taken due to the risk of over sedation when opioids and benzodiazepines are co-administered. Conversely, when benzodiazepines are reduced rapidly, withdrawal, agitation, and other potentially life-threatening side effects such as seizures may occur. The use of a flexible patient-centered approach is important where weaning of multiple medications is needed [65].

## 6. Adjuvant Therapies for Opioid Reduction Therapy

When implementing opioid-reduction pharmacological treatments, adjuvant therapies are regularly considered for possible integration into treatment regimens. Adjuvant analgesics target specific aspects of nociceptive and neuropathic pain physiology, reducing pain and opioid consumption [25]. Nociceptive analgesics include acetaminophen, non-steroidal anti-inflammatory drugs (NSAIDS), and glucocorticoids. Neuropathic analgesics commonly include gabapentinoids, lidocaine, ketamine, alpha-2-agonists, and rare use of tricyclic antidepressants and serotonin and norepinephrine reuptake inhibitors. Low dose ketamine may be helpful as an adjuvant to assist in weaning off opioid therapy [66].

Cannabidiol (CBD) is another adjuvant therapy that is increasingly considered for chronic pain management treatments. CBD is one of the active cannabinoids identified in cannabis. CBD does not appear to have the hallucinogenic/intoxication side effects of another major component of cannabis, tetrahydrocannabinol (THC) [67]. Randomized controlled studies are needed to further examine the efficacy of medical cannabis and its potential use in opioid weaning therapy.

Integrating adjuvant therapies with nonpharmacological therapies such as cognitive behavioral therapy, mindfulness, hypnosis, and acupuncture is commonly employed during opioid-reduction pharmacological treatments to optimize patient comfort, functional well-being, and quality of life. Consultation with a pain management specialists can facilitate this integrative opioid reduction approach.

## 7. Conclusions

Opioid use has risen significantly in the past two decades. Although opioids are an effective means of managing acute pain, risks associated with opioids are plentiful and may outweigh such benefits, especially when treating chronic pain. Having an accurate understanding of opioid-related side effects, age appropriate tools for assessment of withdrawal, and opioid reduction strategies, may help to minimize symptoms of withdrawal and safely reduce or discontinue opioid therapy. 

As outlined above, opioids can be safely tapered in both acute and chronic settings by gradually reducing the total opioid dose or, if appropriate, transitioning to a long acting opioid such as methadone, followed by a gradual weaning protocol and close observation for symptoms of withdrawal. Opioid-sparing adjuvants, including acetaminophen, non-steroidal agents, gabapentinoids, benzodiazepines, ketamine and alpha-1-agonists, can also provide safe and effective analgesia and sedation, and may reduce the necessity and/or dose of opioid therapy. Alpha-1-agonists in particular may help in management of withdrawal symptoms. Opioid reduction utilizingpharmacological protocols provides a safe and efficacious way to decrease opioid therapy when this is appropriate, and minimizes related adverse side effects.

## Figures and Tables

**Table 1:** Summary of physiological effects [29,30,31].

Term	Definition	Causative Mechanism
**Dependence**	A physiologic and biochemical adaptation of neurons such that removing a drug precipitates withdrawal or abstinence syndrome	Activation of second-messenger protein kinases; changes in neurotransmitter levels; changes in neuronal networks
**Withdrawal**	A clinical syndrome that manifests after stopping or reversing a drug after prolonged exposure to that drug	Superactivation of AC; opioid receptor coupling to G_s_ protein; activation of excitatory amino acid receptors
**Tolerance**	Decreasing clinical effects of a drug after prolonged exposure to it	Upregulation of the cAMP pathway; desensitization of opioid receptors
**Addiction**	A chronic, relapsing syndrome of psychological dependence and craving a drug for its psychedelic, sedative, or euphoric effects; characterized by compulsion, loss of control, and continued use of a substance despite harmful effects	Activation of dopaminergic reward systems in nucleus accumbens; mechanisms associated with tolerance and dependence

**Table 2:** Sample Opioid Weaning Schedules.

Weaning schedules	
IV morphine dose	Methadone total daily dose
<0.05 mg/kg/h	0.3 mg/kg/day
0.05–0.1 mg/kg/h	0.4 mg/kg/day
0.11–0.2 mg/kg/h	0.6 mg/kg/day
0.21–0.4 mg/kg/h	0.8 mg/kg/day
>0.4 mg/kg/h	1 mg/kg/day
IV fentanyl dose	Methadone total daily dose
1 µg /kg/h	0.05 mg/kg/day
2 µg/kg/h	0.1 mg/kg/day
3 µg/kg/h	0.15 mg/kg/day
4 µg/kg/h	0.2 mg/kg/day
